# Self-Amplifying RNA Vaccines After the First Approvals: Technology, Development Landscape, and Regulatory Science Considerations

**DOI:** 10.3390/ijms27219457

**Published:** 2026-10-23

**Authors:** Sunghyun Yi, Eunhye Seo, Kee-Jong Hong, Pil-Gu Park

**Affiliations:** 1 Korea mRNA Vaccine Initiative, Gachon University, Seongnam 13120, Republic of Korea; yifromseoul@gmail.com (S.Y.); graceseo@gachon.ac.kr (E.S.); 2 Department of Health Policy and Management, General Graduate School, Gachon University, Seongnam 13120, Republic of Korea; 3 Department of Microbiology, College of Medicine, Gachon University, Incheon 21199, Republic of Korea; 4 Department of Life Sciences, Gachon University, Seongnam 13120, Republic of Korea

**Keywords:** self-amplifying RNA, mRNA vaccines, vaccine regulation, infectious disease vaccine, cancer vaccine, translational development

## Abstract

Self-amplifying RNA (saRNA) is a next-generation nucleic acid vaccine platform that enhances antigen expression through intracellular amplification of replicon RNA without producing infectious virions. saRNA constructs encode an alphavirus-derived replicase comprising nonstructural proteins nsP1–nsP4 and can elicit robust immune responses at low RNA doses. Replication intermediates may activate RIG-I, MDA5, and endosomal TLR3/7/8, contributing adjuvant-like activity. Kostaive (ARCT-154; zapomeran) became the first saRNA vaccine to receive full marketing approval in Japan in 2023, followed by authorization in the European Union in 2025 and the United Kingdom in 2026, demonstrating the platform’s clinical and regulatory feasibility. Development now extends beyond COVID-19 to seasonal and pandemic influenza, rabies, Crimean–Congo hemorrhagic fever, Nipah virus disease, and individualized cancer vaccines. However, the longer transcripts and additional replicase and subgenomic-promoter elements make in vitro transcription efficiency, full-length RNA content, double-stranded and truncated RNA impurities, lipid nanoparticle encapsulation, and potency testing particularly important. Regulatory assessment increasingly focuses on correct replicase and antigen expression, the magnitude and duration of self-amplification, biodistribution and persistence, and comparability. This review synthesizes the scientific basis, development landscape, manufacturing challenges, and regulatory science considerations of saRNA vaccines.

## 1. Introduction

The COVID-19 pandemic provided decisive clinical validation of mRNA vaccine technology. BNT162b2 from Pfizer–BioNTech and mRNA-1273 from Moderna were developed rapidly, achieved high protective efficacy, and were deployed globally on an unprecedented scale [1,2,3]. However, depending on the product and indication, conventional mRNA vaccines use RNA doses in the tens of micrograms, and the first licensed products required low-temperature distribution while also presenting challenges related to large-scale manufacturing and raw-material costs [1,2,3,4]. In addition, mRNA expression is transient, and vaccination against emerging variants has involved repeated booster doses, sustaining interest in next-generation RNA platforms with greater dose-sparing potential and improved supply accessibility [5,6].

Self-amplifying RNA (saRNA) is a replicon RNA platform that extends the conventional mRNA architecture. saRNA contains alphavirus-derived genes encoding the nsP1–nsP4 replicase, which amplifies genomic-length RNA and antigen-encoding subgenomic RNA in the cytoplasm, thereby increasing antigen expression [7,8,9]. Consequently, saRNA has the potential to elicit comparable or stronger humoral and cellular immune responses at a lower RNA mass dose than conventional mRNA [10,11,12]. Its adjuvant-like activity arises in part from double-stranded RNA (dsRNA) replication intermediates generated during intracellular amplification, which are recognized as non-self by cytosolic RNA sensors such as RIG-I, MDA5, and PKR and thereby activate innate immunity. Excessive type I interferon signaling, however, can suppress translation through PKR-mediated eIF2α phosphorylation and the OAS/RNase L pathway; sequence design, purification, and formulation therefore require careful optimization [13,14].

Clinical development of saRNA vaccines accelerated in the early 2020s. ARCT-154 (Kostaive; zapomeran), originated by Arcturus Therapeutics and developed with regional and commercial partners, received full marketing approval in Japan in 2023, followed by authorization in the European Union in 2025 and the United Kingdom in 2026 [15,16,17,18,19,20]. This progression marked an important milestone demonstrating the technical and clinical feasibility of the platform. Building on these advances, clinical applications of saRNA are expanding beyond COVID-19 to seasonal influenza, H5N1 avian influenza, rabies, Crimean–Congo hemorrhagic fever, and cancer vaccines [21,22,23,24,25,26,27,28,29,30,31,32,33,34,35,36,37,38,39,40,41,42,43,44,45,46,47,48,49,50,51,52]. Nevertheless, compared with conventional mRNA, saRNA presents distinctive manufacturing, quality, and nonclinical challenges because of its longer transcript and replicase-coding region. These include in vitro transcription (IVT) efficiency, control of dsRNA impurities, lipid nanoparticle (LNP) encapsulation efficiency, potency specification, and assessment of biodistribution and persistence associated with intracellular amplification [9,16,53,54].

This review describes the molecular design principles, self-amplification mechanism, and immunological properties of saRNA vaccines and synthesizes major preclinical and clinical developments. It further examines key research and development challenges, including manufacturing, quality control, and regulatory science considerations, and discusses future directions such as variant updating, preparedness for emerging infectious diseases, expansion into cancer immunotherapy, and improved vaccine access.

## 2. Self-Amplifying RNA Platform Technology

### 2.1. Molecular Architecture and Design Principles

Like conventional mRNA vaccines, saRNA contains a 5′ cap, 5′ untranslated region (UTR), coding sequence, 3′ UTR, and poly(A) tail. It is distinguished by the additional presence of a replicase open reading frame (ORF) with RNA-dependent RNA polymerase (RdRp) activity and a subgenomic promoter (sgP) [9]. The replicase is generally derived from the alphavirus nonstructural proteins nsP1–nsP4, including those of Venezuelan equine encephalitis virus (VEEV), Sindbis virus, and Semliki Forest virus (SFV) [55,56]. Structural-protein genes are removed so that the construct is designed as a noninfectious RNA platform that cannot produce infectious virions. Nonetheless, because alphavirus backbones are used, recombination with circulating viruses has been considered a theoretical risk; construct design and characterization should therefore minimize sequence identity and evaluate recombination and the potential generation of replication-competent chimeric viruses [57].

**Figure 1:**
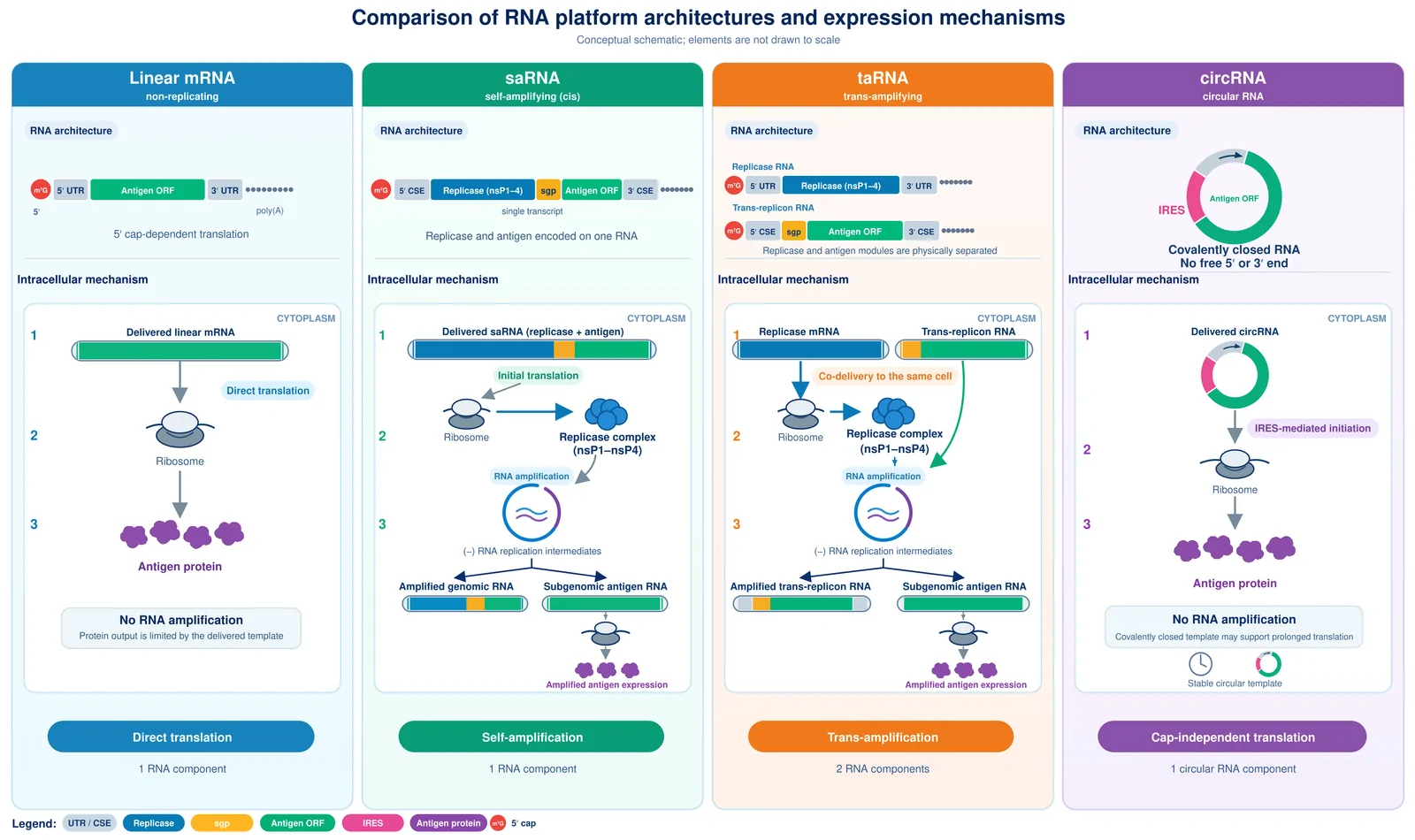
Comparison of RNA platform architectures and expression mechanisms. Linear mRNA undergoes direct translation without intracellular RNA amplification. In cis self-amplifying RNA (saRNA), replicase and antigen are encoded on a single RNA; replicase-generated negative-strand intermediates template genomic and subgenomic RNAs, amplifying antigen expression. Trans-amplifying RNA (taRNA) separates replicase and antigen modules into two co-delivered RNAs. Circular RNA (circRNA) supports cap-independent translation from a covalently closed template without replicase-mediated RNA amplification. Numbered steps indicate the sequence of intracellular events; arrows denote translation or replicase-mediated RNA synthesis. Dotted 3′ tails represent poly(A). The schematic is conceptual and not drawn to scale. Abbreviations: CSE, cis-acting sequence element; IRES, internal ribosome entry site; nsP, nonstructural protein; ORF, open reading frame; sgP, subgenomic promoter; UTR, untranslated region.

Transcript length in conventional mRNA vaccines varies with the antigen and UTR design, and full-length transcripts encoding the SARS-CoV-2 spike protein can extend to several kilobases. In alphavirus-derived saRNA, the replicase region alone is approximately 7 kb, and the addition of an antigen ORF typically produces a longer transcript of approximately 7–12 kb [8,9]. This increased length can reduce the yield of full-length transcripts during IVT, increase the formation of truncated RNA and dsRNA by-products, and complicate LNP encapsulation and stability control [8,9,13,53,54]. When appropriately designed, purified, and formulated, however, saRNA can induce immune responses comparable to or greater than those elicited by conventional mRNA at doses of approximately 0.1–10 μg, offering potential advantages in raw-material use and manufacturing output [10,11,12,18]. Table 1 provides the platform comparison, while Figure 1 offers a visual overview of saRNA architecture and the sequential amplification mechanism alongside the other RNA platforms.

### 2.2. Mechanism of Self-Amplification

Following uptake in a delivery vehicle such as an LNP, saRNA is released into the cytoplasm primarily through endocytosis and endosomal escape. The 5′-proximal replicase ORF is translated first, generating an nsP1–nsP4 polyprotein that undergoes proteolytic processing to form the replication complex. This complex uses the input positive-sense saRNA as a template to synthesize negative-strand RNA [5,9,56,60]. The negative strand then serves as a template for full-length genomic RNA and antigen-encoding subgenomic RNA. Because the sgP drives abundant transcription of the antigen ORF, multiple subgenomic RNA molecules can be generated from a single-input saRNA molecule, thereby amplifying antigen expression. Intracellularly synthesized antigen is predominantly presented through major histocompatibility complex (MHC) class I, promoting CD8+ T-cell responses. Secreted antigen or antigen released during cell death can be taken up by neighboring antigen-presenting cells and displayed through the conventional exogenous MHC class II pathway to activate CD4+ T cells; a fraction can also enter cross-presentation pathways and further contribute to CD8+ T-cell responses [5,56,61,62].

One quantitative analysis of genomic and antigen-containing RNA used a VEEV-derived luciferase construct in 293T cells. Casmil et al. reported that duplex reverse-transcription droplet digital PCR (RT-ddPCR) detected 19–108-fold more copies of the antigen-coding region than of the nsP3 region over 4–67 h after transfection [63]. Since the antigen-coding assay detects both full-length genomic RNA and sgP-derived subgenomic RNA, the result is best interpreted as preferential accumulation of antigen-containing RNA.

At the protein expression level, Vogel et al. compared 4 μg of unformulated luciferase saRNA and conventional mRNA in mice and observed a delayed saRNA peak around day 8 with approximately fivefold higher peak bioluminescence [10]. Taken together, these studies provide quantitative evidence that self-amplification can increase antigen-region RNA abundance and reporter output under defined experimental conditions. They do not, however, establish a universal subgenomic-to-genomic RNA ratio or a fixed fold increase in vaccine-antigen production, because the assays measured regional RNA copies and reporter activity rather than isolated RNA species or absolute antigen mass, and the results depend on construct design, formulation, sampling time, and dose normalization.

Single- and double-stranded RNA replication intermediates generated during self-amplification can activate cytosolic pattern-recognition receptors such as MDA5 and RIG-I and, depending on cellular context, endosomal TLR3, TLR7, and TLR8, resulting in type I interferon and proinflammatory cytokine responses [13,14]. These innate signals can enhance antigen presentation and favor a T helper 1 (Th1)-biased immune response, but excessive interferon signaling may suppress mRNA translation and reduce antigen expression. Accordingly, saRNA design requires co-optimization of replicase activity, UTR and sgP elements, innate immune evasion and translation, the decision whether and how to use modified nucleosides, removal of dsRNA impurities, and LNP composition. One experimental approach co-delivered non-replicating mRNAs encoding the vaccinia virus immune-evasion proteins E3, K3, and B18, rather than administering those proteins directly, to enhance saRNA expression [64].

Whether saRNA vaccination induces anti-replicase immunity and affects subsequent use of the same replicase backbone has been examined mainly in preclinical models. In female mice, saRNA–LNP vaccination induced adaptive responses against the VEEV-derived replicase, including replicase-specific IgG and IFN-γ-producing CD4+ and CD8+ T cells. Pre-existing anti-replicase immunity altered subsequent saRNA expression kinetics, with lower expression during the first few days but more prolonged expression at later time points, and reduced antigen-specific Th1 responses; protection against H5N1 challenge nevertheless remained intact [65]. The EMA assessment of Kostaive separately noted the theoretical possibility that pre-existing anti-replicase T-cell immunity from natural alphavirus infection or live alphavirus vaccination could affect immunogenicity and that vaccine-induced anti-vector responses could influence repeat administration [16].

Human evidence remains limited but is no longer absent. In a 2026 study of a VEEV-derived viral replicon particle (VRP)–srRNA HER2 vaccine, nsP-specific IFN-γ T-cell responses were detected in 7 of 18 patients after three doses administered at weeks 0, 2, and 4; the responses were heterogeneous, generally peaked at weeks 6–8, and declined by month 3 [66]. The same study measured VRP-neutralizing antibodies, which primarily reflect immunity to viral particle structural proteins rather than replicase-specific antibodies. Because VRP delivery incorporates VEEV structural proteins, these findings are not directly equivalent to those expected with LNP-delivered saRNA vaccines. By contrast, the ARCT-154 phase 3 booster trial and its 12-month follow-up evaluated SARS-CoV-2 antigen-specific immunogenicity after a single ARCT-154 booster in participants who had previously received conventional mRNA vaccination; neither report included replicase-specific immune assays or serial exposure to the same saRNA replicase backbone [18,19]. The mouse LNP-saRNA study cited above did not assess cross-protection against or enhanced susceptibility to circulating alphaviruses, so these implications remain unresolved [65]. Prospective replicase-specific immune monitoring may therefore be considered in clinical programmes planning repeated use of a common replicase backbone.

### 2.3. Platform Variant: Trans-Amplifying RNA

Trans-amplifying RNA (taRNA) is being developed as a derivative of the saRNA concept. A common taRNA design partitions these functions between a non-replicating mRNA encoding the replicase and an antigen-containing trans-replicon with the required cis-acting elements but no complete functional replicase ORF. This avoids manufacturing a single very long transcript and allows the shorter antigen-containing RNA to be optimized separately, potentially improving IVT efficiency and process flexibility [59].

Separating the replicase and antigen cassettes can increase design flexibility and manufacturability, but the two RNAs must be co-delivered to the same cell at an appropriate ratio. The original proof-of-concept study administered an unformulated mixture of the two RNAs intradermally and therefore did not establish that both RNAs were loaded into a common LNP [59]. As of May 2026, taRNA remained predominantly at the preclinical stage, and no product had received authorization for human use.

For lipid-based taRNA delivery, co-formulating both RNAs before particle formation and mixing two separately formulated RNA-particle populations are distinct strategies. Neither a bulk RNA ratio nor total encapsulation efficiency alone establishes same-cell delivery or co-loading of every particle. In supporting experiments with two conventional reporter mRNAs, co-encapsulation in lipoplexes improved single-cell ratiometric expression compared with mixing separately loaded lipoplexes [67]. This was not a taRNA-LNP trial. It supports testing RNA-specific encapsulation, intracellular co-delivery, and functional trans-amplification for the selected taRNA formulation, rather than assuming that either loading strategy is universally optimal.

### 2.4. Lipid Nanoparticle Delivery Systems

Efficient intracellular delivery is essential for saRNA, and many clinical-stage candidates use LNP- or nanostructured lipid carrier (NLC)-based formulations [9,68]. LNPs generally comprise an ionizable lipid, a helper or structural lipid such as DSPC, cholesterol, and a PEG–lipid [4,9], and deliver RNA to the cytoplasm through endocytosis followed by endosomal escape. Because saRNA transcripts are long, reproducible control of particle size, polydispersity index (PDI), encapsulation efficiency, RNA integrity, residual solvents and lipid impurities, and stability is particularly important [9,16,54].

Kostaive (ARCT-154; zapomeran) is formulated in an ATX-126-containing LNP and was authorized at 5 μg of RNA per dose after demonstrating clinical immunogenicity [16,17,18,69], underscoring the importance of delivery-system design to saRNA vaccine performance. Other studies have investigated lyophilized ionizable LNPs incorporated into microneedle array patches [70] and lyophilized NLC formulations capable of maintaining long-term stability at room temperature [68]. Such approaches could reduce dependence on cold-chain logistics and improve vaccine access in resource-limited settings.

Direct stability comparisons between saRNA and conventional mRNA remain limited, and the available evidence is formulation-specific. In matched formulations, Ly et al. found that maintaining RNA integrity and encapsulation was more challenging for the longer saRNA than for conventional mRNA, with outcomes dependent on lipid composition and processing conditions [58]. Interpreting such comparisons requires distinguishing physicochemical stability during storage from the duration of intracellular expression; the prolonged expression conferred by self-amplification arises from replicase activity after delivery and does not indicate greater intrinsic resistance to degradation [10,58]. On this basis, the thermostability reported for a lyophilized saRNA–NLC formulation is best read as product-specific evidence that formulation design can offset the stability penalty associated with longer transcripts, rather than as a general stability advantage of saRNA over conventional mRNA [68].

### 2.5. Preclinical Characteristics

Although immune responses vary with antigen sequence, replicon backbone, and delivery formulation, preclinical studies have generally supported saRNA as a dose-sparing vaccine platform capable of eliciting robust humoral and cellular immunity at low RNA doses. Vogel et al. compared influenza hemagglutinin-encoding saRNA with synthetic mRNA and showed that 1.25 μg of saRNA could provide protection comparable to that achieved with 80 μg of mRNA. Challenge protection was observed with saRNA constructs encoding H1N1, H3N2, or influenza B antigens individually and with a trivalent saRNA combination, supporting the applicability of saRNA to multivalent vaccine design [10]. Studies using SARS-CoV-2 antigens have likewise repeatedly demonstrated dose-sparing potential and Th1-biased immunity. McKay et al. reported that an LNP-formulated saRNA encoding prefusion-stabilized SARS-CoV-2 spike induced dose-dependent anti-spike IgG and pseudovirus and wild-type virus neutralization in mice across a 0.01–10 μg dose range, together with a Th1-biased response indicated by IgG subclasses and splenocyte IFN-γ responses [12].

The AAHI-SC2 saRNA/NLC vaccine described by Voigt et al. elicited neutralization of Wuhan, Alpha, Beta, and Delta pseudoviruses, generated bone marrow-resident IgG- and IgA-secreting cells, and induced an IFN-γ-dominant Th1-biased T-cell response in C57BL/6J mice. Its lyophilized formulation retained immunogenicity after 6 months at room temperature and 10 months under refrigeration, demonstrating thermostability [68]. In a study extending to nonhuman primates, the LION-formulated alphavirus replicon RNA vaccine LION/repRNA-CoV2S induced a Th1-type IgG response after a single dose in mice and antigen-specific T-cell responses in the lung and spleen after prime–boost vaccination. In nonhuman primates, both prime-only and prime–boost regimens induced robust antibody responses and limited but measurable T-cell responses, with neutralizing antibodies persisting for at least 70 days [71]. Collectively, these studies illustrate low-dose activity, Th1-skewed cellular immunity, and antibody durability in selected preclinical saRNA systems; the AAHI-SC2 study additionally demonstrates that formulation-specific thermostability can be achieved with a lyophilized saRNA–NLC product.

Cellular immunity has received particular attention in preclinical saRNA work. In mice, saRNA constructs encoding the conserved influenza nucleoprotein (NP) and matrix protein 1 (M1), delivered either separately or in a bicistronic vector, elicited polyfunctional CD4+ Th1 responses, with NP-containing constructs additionally inducing cytotoxic CD8+ responses and protection against homologous and heterosubtypic challenge [72]. Route-dependent pulmonary resident memory T-cell responses have also been reported for a dendron-based nanoparticle saRNA formulation [62]. Depletion experiments indicate that these responses can be protective in their own right: in K18-hACE2 mice vaccinated with an saRNA SARS-CoV-2 vaccine, B-cell depletion before vaccination did not abolish protection of lung and brain, whereas T-cell depletion immediately before challenge permitted substantial infection [16]. On this basis, saRNA is a reasonable platform to evaluate against pathogens for which cellular immunity contributes to control.

Whether saRNA is more T-cell dominant than conventional mRNA is a separate question, and the available matched comparison does not support that framing. In mice, ARCT-021 and the non-amplifying mRNA construct ARM3013, which encoded the same spike antigen, were compared at 0.2, 2, and 10 μg. Spike-specific IgG in the 10-μg conventional mRNA group was similar to that in the 0.2-μg ARCT-021 group, which the EMA assessment interpreted as an approximately 50-fold difference in dose potency; neutralizing antibodies were detected at the 2- and 10-μg ARCT-021 doses but not in the conventional mRNA groups [16]. The study also reported differences in effector-memory CD8+ populations, Th1/Th2 balance, and IFN-γ ELISpot responses. Thus, saRNA enhanced both humoral and cellular readouts in this mouse study rather than demonstrating selective T-cell dominance.

These potential benefits must be balanced against observed reactogenicity and development risks. Kostaive trials reported local and systemic reactions, including injection-site pain, fatigue, headache, and myalgia; low RNA mass should not be interpreted as absence of reactogenicity [16,18]. Excessive innate signaling can also inhibit translation, while long-transcript manufacture increases the analytical burden [14,54]. Anti-replicase immunity and theoretical recombination warrant product-specific investigation, but should not be presented as established clinical harms of licensed saRNA vaccines [16,65,69].

## 3. Research and Development Landscape

### 3.1. COVID-19 Vaccines

Clinical development of saRNA vaccines against COVID-19 expanded rapidly from the early 2020s, with multiple candidates entering human trials. ARCT-154 (Kostaive; zapomeran), developed by Arcturus Therapeutics with regional clinical, regulatory, and commercial partners, became the first saRNA vaccine to receive full marketing approval. Evidence for its safety, immunogenicity, and efficacy was generated through phase 1, 2, 3, and 3b trials in Vietnam and comparative booster studies in Japan [16,17,18,19]. It received manufacturing and marketing approval in Japan in November 2023 [15,17], European Commission marketing authorization in February 2025 [16], and authorization from the UK Medicines and Healthcare products Regulatory Agency in January 2026 [20]. The publicly disclosed clinical development landscape is summarized in Table 2.

Gennova Biopharmaceuticals in India developed GEMCOVAC-19 using HDT Bio’s LION/repRNA technology and received emergency use authorization from the Indian regulator in June 2022 [27]. The company subsequently completed a phase 2/3 trial of the Omicron-adapted GEMCOVAC-OM (CTRI/2022/10/046475), obtained an additional emergency authorization in June 2023, and reported safety and immunogenicity as a heterologous booster [24,28]. In the United Kingdom, Imperial College London and VaxEquity evaluated the low-dose LNP-nCoVsaRNA candidate COVAC1 in phase 1 and 2a studies; however, seroconversion rates in the initial study were lower than anticipated, and no later-stage development was identified in the public sources reviewed through 6 May 2026 [25,26].

VLPCOV-02, developed by VLP Therapeutics Japan, is a modified-base saRNA incorporating 5-methylcytosine (5mC) and was designed to attenuate excessive innate immune activation and reduce reactogenicity [73]. Interim phase 1 data indicated fewer febrile events than with VLPCOV-01 and favorable tolerability [73], and Part 2 results published in 2026 reported RBD-specific IgG, neutralizing antibodies, and CD4+ and CD8+ T-cell responses at doses of 3 and 7.5 μg [30]. VLP Therapeutics Japan also conducted phase 3 studies in Japan of VLPCOV-04, an Omicron XBB.1.5 booster candidate [29].

### 3.2. Indications Beyond COVID-19

saRNA development is extending beyond COVID-19 to vaccines against a range of infectious diseases. In influenza, Pfizer and BioNTech completed a phase 1 dose-finding study of seasonal influenza saRNA vaccine preparations (NCT05227001) [31,32]. Arcturus and CSL completed a phase 1 study of the multivalent seasonal influenza candidate ARCT-2138 (NCT06125691), and a clinical study report was finalized in 2025 [33,34]. ARCT-2304 (LUNAR-H5N1), an Arcturus saRNA candidate targeting H5N1 avian influenza, entered a US phase 1 study (NCT06602531) and received FDA Fast Track designation in 2025 [35,36]. GSK’s rabies candidate RG-SAM was evaluated in a phase 1 study (NCT04062669) [47], whereas Replicate Bioscience’s self-replicating RNA rabies candidate RBI-4000 reported safety, immunogenicity, and durability in a phase 1 study spanning doses of 0.1–10 μg (NCT06048770) [11,43,44].

For emerging infectious diseases, HDT Bio initiated a phase 1 study of HDT-321 against Crimean–Congo hemorrhagic fever virus (NCT06799013) [39], and Gennova, with support from the Coalition for Epidemic Preparedness Innovations, is developing an saRNA vaccine candidate against Nipah virus disease [51]. Within the COVID-19 field, the Access to Advanced Health Institute and ImmunityBio evaluated the NLC-formulated saRNA booster candidates AAHI-SC2 and AAHI-SC3 in a phase 1/2 study in South Africa (NCT05370040) [40]. In oncology, Gritstone Bio’s individualized neoantigen vaccine regimen GRANITE, which combines a ChAd68 prime with an saRNA boost, accumulated phase 1/2 clinical experience in advanced solid tumors [52]. The company’s saRNA candidates encoding COVID-19 T-cell epitopes, including GRT-R910 and GRT-R912/R914/R918, were also evaluated clinically [41,42].

## 4. Challenges and Future Directions

### 4.1. Manufacturing and Quality Control

Commercialization of saRNA vaccines requires reliable manufacture of long RNA transcripts and rigorous quality control. Although the length of conventional mRNA varies by antigen, alphavirus-derived saRNA is typically 7–12 kb because of the replicase ORF, increasing the risk of reduced full-length RNA yield, truncated transcripts, dsRNA by-products, and template-related impurities during IVT [8,9,13,53,54]. Because dsRNA impurities can stimulate innate pathways and inhibit translation, purification must control manufacturing-derived dsRNA and short RNA species [13,14,54]. These impurities are distinct from replication intermediates generated after cellular delivery.

Validation of analytical methods for RNA integrity and purity is another central challenge. Critical quality attributes that can directly affect antigen expression and safety include 5′-capping efficiency, poly(A)-tail length, the proportion of full-length RNA, RNA content and identity, residual DNA template, residual enzymes, and endotoxin [54,74]. Full-length RNA control is especially important for saRNA because both the replicase ORF and antigen ORF must remain intact for self-amplification. Routine release potency testing should be distinguished from mechanistic characterization of replicase expression, RNA amplification, and antigen production. The latter need not all be measured by a single release assay [16,54].

During construct and process development, representative analytical methods include sequence analysis for identity, capillary or agarose gel electrophoresis and chromatography for RNA size distributions, liquid chromatography for cap and poly(A) characterization, dsRNA-specific immunoassays, and quantitative PCR for residual DNA [16,54]. An saRNA-specific example is duplex one-step RT-ddPCR with linkage analysis of distant regions, which helps distinguish linked transcripts from coincident fragments; it complements, rather than replaces, size-based integrity and functional testing [63]. For process interpretation, total RNA yield should be considered together with an orthogonal measure of full-length RNA or RNA integrity, because total mass alone does not distinguish intact transcripts from fragmented RNA.

For the LNP drug product, particle size, PDI, RNA encapsulation efficiency, zeta potential, morphology, lipid composition, lipid-related impurities, residual solvents, oxidation and degradation products, and storage stability should be controlled reproducibly [9,16,54]. The long saRNA transcript can affect encapsulation and particle uniformity, making optimization of microfluidic mixing conditions, the nitrogen-to-phosphate ratio, ionizable lipid structure, helper-lipid composition, and lyophilization conditions essential [9,68,70]. Long-term and accelerated stability studies should assess RNA fragmentation, LNP aggregation or leakage, and loss of potency under relevant storage conditions [16,54].

During formulation development and finished-product control, dynamic light scattering assesses size and PDI, while a fluorescence assay before and after particle disruption estimates unencapsulated and total RNA. RNA extracted from particles should also be assessed for integrity, since high encapsulation efficiency alone does not establish that the payload is intact. Lipid composition and degradation products can be assessed chromatographically [54]. A representative saRNA formulation study evaluated size, encapsulation, RNA integrity, and cellular reporter expression together [58]. A product-specific antigen-expression or biological-activity assay is needed to connect physicochemical attributes to function. Kostaive illustrates the importance of this distinction: the EMA required additional validation and supporting batch data for its ELISA-based finished-product potency assay [16].

Industrial experience with commercial-scale IVT, purification, and LNP encapsulation remains more limited for saRNA than for conventional mRNA. The approval of Kostaive provided an important regulatory precedent showing that a good manufacturing practice process and comparability package could be implemented at a commercial scale. During transition from clinical to commercial manufacturing, comparability of pre- and post-change processes and consistency of RNA and LNP quality attributes were central regulatory considerations [16,17]. This experience suggests that platform-level comparability strategies and the use of prior knowledge, alongside batch-specific evidence, will become increasingly important as future saRNA candidates enter clinical development and licensing pathways [54].

The quality-assurance timeline is stage-based rather than using a universal number of days. Exploratory assays and process controls are established during preclinical and early clinical development; they are then qualified or validated for their intended use as development progresses; commercial process consistency is demonstrated before marketing authorization. WHO distinguishes this from preliminary trials, for which full process validation is not generally expected, although aseptic processing and sterility control must already be demonstrated [74]. Release testing is performed on each batch against the applicable drug-substance and finished-product specifications before use; the exact panel and any justified test omission are product-specific [54,74]. We did not identify a universal saRNA batch-release turnaround time in these guidelines or in the publicly available Kostaive assessment report.

Stability-indicating assays should be established during development using stressed material and followed in real-time studies throughout the proposed shelf life. Relevant studies include intermediate hold times, transport conditions, freeze–thaw exposure when applicable, and the in-use period after reconstitution. RNA integrity, encapsulation or leakage, particle changes, and potency should be followed at justified time points. Manufacturing-site, scale, process, or formulation changes trigger comparability assessment and, where necessary, renewed validation or stability studies [16,54,74]. Such changes are managed within the product’s lifecycle framework [75]. These are representative control strategies, not universal numerical specifications; assay performance, acceptance limits, and sampling schedules require product-specific justification.

### 4.2. Regulatory Science Considerations

#### 4.2.1. General Considerations

Like conventional mRNA vaccines, prophylactic saRNA vaccines can generally be regulated as biological products or vaccines for the prevention of infectious diseases. Their self-amplifying function, however, introduces distinctive characteristics that warrant additional quality and nonclinical assessment [54,74]. The European Medicines Agency’s 2025 draft guideline on the quality aspects of mRNA vaccines explicitly allows saRNA to fall within the scope of prophylactic mRNA vaccines and, in addition to drug substance and drug product data shared with non-amplifying mRNA, calls for evidence of correct replicase and antigen expression, phenotypic self-amplification, and a description of the relevant sequence elements [54]. WHO Technical Report Series 1039, Annex 3, also provides recommendations for evaluating the quality, safety, and efficacy of mRNA vaccines against infectious diseases and can inform global regulatory strategies for saRNA candidates [74].

Because saRNA undergoes intracellular RNA amplification, nonclinical assessment should characterize whether and for how long relevant RNA species and the encoded antigen persist in intended and unintended tissues. The general biodistribution principles in ICH S12 may be informative when platform-specific persistence warrants characterization, although prophylactic vaccines are outside the formal scope of ICH S12 and prophylactic saRNA vaccines are generally regulated as vaccines rather than gene therapy products [76]. Regulatory assessments have therefore emphasized not only the tissue distribution of the RNA and delivery-system components but also biodistribution data that characterize the duration of self-amplification and patterns of persistence and clearance after administration [16,17,69].

Regulatory approaches also differ in the degree of surveillance applied to vaccine-related immune-mediated safety events, notably vaccine-associated enhanced disease (VAED) and vaccine-associated enhanced respiratory disease (VAERD), because of the novelty of the platform. Some regulators have considered these risks low on the basis of nonclinical immune-response profiles, whereas others have required active monitoring through postmarketing risk-management plans in view of limited early clinical exposure. For future candidates using the same platform, the balance between nonclinical evidence and postmarketing surveillance is therefore likely to remain an important review issue [16,17,69].

With alphavirus-based saRNA, the theoretical possibility that recombination with a circulating alphavirus could generate a replication-competent chimera has been considered in regulatory assessment. Recent experimental work identified rare chimeric constructs in vitro but found that mechanisms including superinfection exclusion substantially mitigated the emergence of recombination events in vivo [57]. In its assessment of Kostaive, the MHRA concluded that recombination between the VEEV-derived replicase and a circulating alphavirus could not be excluded theoretically but was unlikely, based on superinfection exclusion, codon optimization, and replicase specificity for the Kostaive RNA construct. The same specificity was also considered to make unintended amplification of a co-administered mRNA vaccine unlikely [69]. Risk mitigation may include minimizing sequence homology between the alphavirus-derived backbone and circulating viruses, using construct designs that reduce recombination potential where applicable, and characterizing the possibility of replication-competent recombinants [57,69].

A distinction is necessary between preauthorization recombination assessment and active surveillance after vaccination. In our review, we did not identify an explicit requirement in the EMA 2025 draft mRNA quality guideline or WHO TRS 1039, Annex 3, for routine molecular surveillance of vaccine recipients for recombinant alphaviruses. The EMA draft addresses replicase and antigen characterization, whereas WHO describes general clinical safety evaluation and postmarketing pharmacovigilance [54,74]. Kostaive-specific review of theoretical recombination risk should therefore not be described as a general requirement for population-wide recombinant-virus testing [16,69].

Future regulatory work could define proportionate, risk-triggered investigations: documenting the construct and relevant exposure context, qualifying assays to distinguish vaccine RNA from potentially replication-competent material, and establishing escalation criteria when an unexpected virological or safety signal occurs. These are proposed research and harmonization priorities, not existing universal release requirements or an indication that recombinant alphaviruses have been detected in vaccine recipients.

Clinical extrapolation from animal challenge models also requires caution. In a nonhuman primate SARS-CoV-2 challenge study, rapid viral clearance occurred even in placebo animals, limiting interpretation of the model’s relevance to human disease [16]. Consequently, challenge protection should be interpreted alongside mechanistic immunogenicity evidence and clinical endpoints rather than used in isolation when translating nonclinical efficacy to humans.

From a product-classification perspective, prophylactic mRNA and saRNA vaccines are generally evaluated as vaccines or biological products; in Europe, vaccines for the prevention of infectious diseases are excluded from the definition of a gene therapy medicinal product [54,74]. For therapeutic saRNA, cancer vaccines, or protein replacement applications, however, the indication and mechanism of action may change the applicable classification, nonclinical package, and potency strategy, which should be clarified through early consultation with the relevant regulator. Platform approaches involving antigen changes, such as variant-updated vaccines or a shared LNP and manufacturing process, may be supported by prior knowledge and comparability data and aligned with ICH Q12 lifecycle management and the EMA’s platform and prior-knowledge concepts [54,75]. As successive variant-adapted candidates such as ARCT-2301 (BA.4/5 bivalent) and ARCT-2303 (Omicron XBB.1.5) are evaluated on the same platform, standardizing comparability packages for antigen updates will be an important determinant of rapid-response capability.

#### 4.2.2. Kostaive (ARCT-154) Assessment Case Study

Kostaive (ARCT-154; zapomeran), the first saRNA vaccine to receive full marketing approval, provides a particularly informative case because the EMA, PMDA, and MHRA assessment documents disclose comparatively detailed immunogenicity, biodistribution, and toxicology data [16,17,69]. The MHRA reviewed the product through the International Recognition Procedure, using the EMA as the reference regulator and broadly accepting its nonclinical conclusions, while conducting a targeted assessment of issues specific to ARCT-154, including the persistence of self-amplification, ATX-126, and the replicase [69]. ARCT-154 and its predecessor ARCT-021 share a VEEV-derived replicase sequence and LNP composition; the difference in the spike antigen cassette allowed direct ARCT-154 data to be interpreted together with supporting ARCT-021 data [16,17,69]. A comparison of the major regulatory findings is provided in Table 3.

After a single 2-μg administration to BALB/c mice, both ARCT-154 and ARCT-021 induced anti-spike protein antibodies and receptor-binding-inhibitory activity against the ancestral strain and the Alpha, Beta, and Gamma variants [17]. In cynomolgus monkeys, two 7.5-μg doses administered 28 days apart were followed by measurement of anti-spike antibodies, receptor-binding-inhibitory activity, neutralizing antibodies, and IFN-γ ELISpot responses. On day 56, neutralizing antibody titers against all strains tested were higher in the ARCT-154 group than in the ARCT-021 group [17]. The EMA and MHRA assessments likewise reported stronger immune responses with ARCT-154 than with ARCT-021 against the ancestral strain and the Alpha, Beta, and Gamma variants, and a Saphire microneutralization assay demonstrated broader neutralization extending to Delta and Omicron BA.1 [16,69]. In a supplementary pharmacology study of ARCT-021, administration of 0.2, 2, or 10 μg to C57BL/6 mice produced a Th1-dominant profile based on IgG subclasses, IFN-γ-producing cells, and Th1/Th2 cytokines [17]. In a dose-ranging comparison, spike-specific IgG elicited by 10 μg of the non-amplifying mRNA construct ARM3013 was reported to be comparable to that elicited by 0.2 μg of ARCT-021 [16]. This comparison involved preclinical candidate constructs and was not a head-to-head comparison with licensed mRNA vaccines such as Comirnaty or Spikevax.

Mouse and nonhuman primate challenge studies with ARCT-021 provided the principal efficacy-supporting data. In K18-hACE2 mice, a single 2- or 10-μg dose administered 30 days before challenge with ancestral SARS-CoV-2 suppressed clinical signs and viral growth in the lung and brain and yielded 100% survival [16,17]. In B-cell and T-cell depletion studies, protection of the lung and brain was maintained after B-cell depletion, whereas infection occurred after T-cell depletion, supporting a contribution of T-cell-mediated immunity to protection [16,69]. In rhesus macaques, ARCT-021 was administered once at 20 or 40 μg or twice at 5 or 20 μg, 28 days apart, followed by intranasal and intratracheal challenge with the ancestral strain [16,17]. The PMDA considered the study to demonstrate suppression of clinical signs and viral growth. The EMA, however, noted rapid viral clearance in untreated animals and no significant difference in TCID50 values between vaccinated and control groups, limiting the model’s relevance to human disease; it therefore treated the study mainly as supportive efficacy and immunogenicity evidence [16,17]. The MHRA’s position under the International Recognition Procedure was broadly consistent with the EMA assessment [69].

Biodistribution and persistence were characterized using complementary studies: a single-dose mouse study of ARCT-021 (active substance mRNA-2002) and its encoded spike protein; a comparative firefly luciferase study of replicon saRNA and non-amplifying mRNA in matched LNPs; and the direct ARCT-154 study ARC24-052 submitted to the MHRA [16,17,69]. In the ARCT-021 study, mRNA-2002 was detectable in plasma and all sampled tissues 2 h after administration and was below the limit of detection in most tissues by day 31, except injection-site muscle and inguinal lymph node. Spike protein became detectable in muscle at approximately 48 h, peaked at 72 h or day 8, and was no longer detectable there by day 15; in the high-dose group, low levels remained detectable in lymph nodes and plasma through day 31 [17]. In the comparative luciferase study, muscle exposure to replicon saRNA was 5.47-fold and 2.85-fold higher than that to non-amplifying mRNA at 10 and 50 μg, respectively, based on area under the concentration–time curve. Amounts were negligible by day 30 for saRNA and by day 15 for non-amplifying mRNA [16,69]. In ARC24-052, ARCT-154 genomic RNA had cleared from plasma, lung, and contralateral muscle by days 8, 15, and 30, respectively; at the injection site, only one of ten animals in the 25-μg group had low-level RNA detected on day 60 [69]. Negative-strand RNA and nsP1 replicase protein were detected only transiently and in a limited distribution. Spike protein had cleared from muscle, spleen, popliteal lymph nodes, and inguinal lymph nodes by days 15, 5, 30, and 60, respectively, supporting a self-limiting amplification profile for ARCT-154 [69].

Repeated-dose good laboratory practice studies with ARCT-154 in rabbits and repeated-dose and developmental and reproductive toxicity studies with ARCT-021 supported the toxicology assessment [16,17,69]. Rabbits received three intramuscular doses of ARCT-154 at 16.75, 25.1, or 33.55 μg at 2-week intervals, followed by a 4-week recovery period. Findings included degeneration and inflammation of skeletal muscle at the injection site, subcutaneous inflammation, transient fever, and inflammation-related clinical pathology changes [16,17]. The EMA and PMDA interpreted these findings as vaccine-induced inflammatory reactions and considered 33.55 μg, the highest dose tested, to be the no-observed-adverse-effect level [16,17]. The MHRA noted attenuated IL-6 and C-reactive protein responses and less pronounced injection-site histopathology with ARCT-154 than with ARCT-021, and accepted the applicant’s explanation that the higher dsRNA content of early ARCT-021 lots may have contributed to the difference in reactogenicity [69]. In the ARCT-021 rabbit developmental and reproductive toxicity study, five doses of 10 or 20 μg were administered from before mating through gestation. Apart from reduced maternal body-weight gain in the high-dose group, no vaccine-related effects on embryo-fetal development or F1 offspring were identified [16,17,69].

The LNP component ATX-126 persisted longer than the mRNA in mice and rabbits and remained detectable through the recovery period in the liver, spleen, injection site, and ovaries in repeated-dose rabbit studies. The EMA, PMDA, and MHRA nevertheless concluded that the findings were not accompanied by adverse histopathology or effects on fertility and that exposure margins relative to clinical use were adequate [16,17,69]. Although a standalone genotoxicity battery was not performed for ATX-126, the PMDA relied on structure–activity relationship analysis and prior use experience, whereas the EMA and MHRA considered an in silico mutagenicity assessment and the ARCT-810 genotoxicity package, which included the structurally related lipid ATX-95; all three agencies considered the genotoxicity risk low [16,17,69]. For VAED and VAERD, the EMA and MHRA concluded that the Th1-skewed and innate immune response did not indicate an increased risk associated with saRNA, whereas the PMDA retained VAED/VAERD as an important potential risk for postmarketing surveillance because of its low incidence and the limited clinical dataset [16,17,69].

### 4.3. Future Research Directions and Outlook

saRNA and related replicon RNA platforms are being evaluated beyond infectious disease vaccines for cancer immunotherapy and therapeutic protein expression [77,78,79]. In oncology, individualized saRNA vaccines can be designed from patient-specific tumor neoantigens. The GRANITE study demonstrated the clinical feasibility of an individualized neoantigen regimen combining a ChAd68 prime with an saRNA boost [52,77]. saRNA has also been proposed for protein replacement therapy in rare diseases, including enzyme-deficiency disorders that may benefit from sustained protein expression [78].

Technical improvement should link molecular optimization to measurable product performance. McGee et al. identified modified nucleotides that, when substituted at 100% in the tested saRNA backbone, suppressed the early interferon response while increasing and prolonging protein expression, and conferred protection against lethal SARS-CoV-2 challenge in mice; notably, N1-methylpseudouridine, the modification used in licensed mRNA vaccines, is not compatible with alphaviral replicase activity, so compatibility cannot be assumed for every nucleotide or replicon [80]. Perkovic et al. first simplified the transreplicon by eliminating the sgP and redesigning the 5′ UTR, and then applied directed evolution to select a faster-replicating variant that increased taRNA expression and antibody responses in mice [81]. Formulation design-of-experiments provides a complementary route to balance integrity, encapsulation, expression, and cellular activation [58]. These approaches should advance through matched comparative studies and product-specific characterization, rather than treating higher reporter expression alone as evidence of improved clinical benefit.

Trans-amplifying RNA and circular RNA (circRNA) are prominent next-generation approaches. taRNA can shorten the antigen-cassette RNA and improve manufacturing flexibility by separating replicase and antigen RNAs, but efficient co-delivery and intracellular stoichiometric control remain central challenges [59]. circRNA has a covalently closed topology that confers resistance to exonuclease-mediated degradation and may support sustained expression. However, cap-independent translation generally requires an internal ribosome entry site or another engineered translation element, and manufacturing, purification, analytical standardization, and translation-efficiency optimization remain unresolved [82,83].

saRNA has strategic value for pandemic preparedness because of its dose-sparing potential and rapid sequence design. ARCT-2304 (LUNAR-H5N1) targets H5N1 avian influenza, HDT-321 targets Crimean–Congo hemorrhagic fever virus, and the Gennova/CEPI Nipah virus program illustrates expansion toward emerging pathogens [35,36,39,51]. The capacity of saRNA to encode multiple antigens or antigenic variants is also being evaluated in multivalent influenza candidates such as ARCT-2138 and PF-07852352. Strategies that co-express multiple antigens from a single plasmid-derived construct or LNP formulation may offer a differentiated route toward broad protection, including future universal influenza vaccines [10,31,32,33,34].

In terms of access and global health equity, the GEMCOVAC series in India and the LNP-nCOV saRNA-02 trial in Uganda illustrate saRNA clinical development in low- and middle-income countries and the potential to establish regional manufacturing capacity [27,28,45,46]. Low RNA mass requirements and thermostable formulations based on NLCs, lyophilization, or microneedle array patches could reduce cold-chain burdens and complement regional deployment strategies [68,70]. These features may also facilitate pandemic stockpiling and rapid distribution.

Beyond LNPs, alternative delivery systems under investigation include NLCs, polymeric nanoparticles, peptide-based carriers, and extracellular vesicles. Tissue-targeted delivery may be particularly relevant to cancer therapy and mucosal vaccination [9,68]. In a preclinical study, intranasal delivery of a replicon-NLC SARS-CoV-2 vaccine induced respiratory mucosal immune responses and protection, supporting further exploration of mucosal saRNA vaccination [84].

Over the next 5–10 years, four developments are likely to shape the field. First, platform-comparability strategies for variant updates and new antigens are expected to become more established through regulatory interaction. Second, clinical programs targeting influenza, Crimean–Congo hemorrhagic fever, Nipah virus, and other emerging infections should provide further evidence for dose sparing in pandemic preparedness. Third, applications beyond prophylactic vaccines, including cancer immunotherapy and protein replacement therapy, are likely to expand. Fourth, if lyophilized, NLC-based, and microneedle-array-patch formulations achieve industrial implementation, they could materially improve vaccine access, including in low- and middle-income countries [5,6,9,68,70,79].

## 5. Conclusions

saRNA is a next-generation RNA platform capable of amplifying antigen expression intracellularly and eliciting strong immune responses at comparatively low RNA doses. The approvals of Kostaive (ARCT-154; zapomeran) in Japan in 2023, the European Union in 2025, and the United Kingdom in 2026 represent major milestones demonstrating the technical and clinical feasibility of the platform [15,16,17,18,19,20]. Preclinical and clinical studies support dose-sparing potential and Th1-biased immunity, but saRNA also presents technical and regulatory challenges distinct from those of conventional mRNA, including long transcript length, dsRNA and short-RNA impurities, LNP optimization, replicase functionality, and assessment of biodistribution and persistence [8,9,13,53,54]. The field now requires robust and standardized analytical methods, scalable manufacturing, well-defined platform comparability strategies, and regulatory frameworks that distinguish platform-level knowledge from product-specific evidence. If these elements advance in parallel, saRNA may extend beyond infectious disease vaccination to cancer immunotherapy and protein replacement applications while contributing to more equitable global access to vaccines.

## Figures and Tables

**Table 1:** Structural features, advantages, and limitations of saRNA and other RNA platforms.

Platform	Structure and Expression Mechanism	Principal Advantages	Principal limitations and Development Risks	Comparison from an saRNA Perspective
Conventional linear mRNA (non-replicating)	A 5′ cap–5′ UTR–antigen ORF–3′ UTR–poly(A) transcript in which the antigen ORF is translated directly in the cytoplasm. It contains no replicase or subgenomic promoter (sgP), and intracellular RNA amplification does not occur.	The architecture and manufacturing process are the simplest, and the transcript is comparatively short. IVT, capping, poly(A) addition, purification, LNP encapsulation, and potency testing are relatively standardized, with the greatest cumulative experience in licensing, use, and regulatory review.	Protein output depends on input RNA, RNA stability, and translation rather than intracellular RNA amplification. In matched preclinical settings, a higher input RNA mass may be required, depending on the antigen and formulation. Physicochemical stability during formulation and storage is formulation-dependent and should be evaluated separately from intracellular expression duration [10,54,58].	saRNA offers amplified expression and dose sparing, whereas linear mRNA provides greater predictability in CMC, analytics, and regulation. Dose sparing should not be equated with fewer required administrations or intrinsically superior storage stability.
Self-amplifying RNA (saRNA)	A single capped and polyadenylated RNA contains an alphavirus-derived replicase ORF (nsP1–nsP4), an sgP, and an antigen ORF. Replicase first synthesizes a negative-strand intermediate from the genomic positive-strand RNA; this intermediate then templates full-length genomic positive-strand RNA and sgP-driven subgenomic antigen RNA.	High antigen expression and immune responses can be achieved at a low RNA dose. RNA replication intermediates may activate innate immune sensing and contribute an adjuvant-like effect. Kostaive was authorized at 5 μg per dose, supporting clinical evidence of dose sparing.	The long transcript makes control of full-length RNA, truncated/short RNA, residual dsRNA and DNA template, capping/poly(A), and LNP particle attributes particularly important. A product-specific potency assay that reflects biological activity must be developed and validated. Excessive type I interferon can suppress translation, and biodistribution, persistence, theoretical recombination, and anti-replicase immunity require additional consideration.	Low RNA mass dose and intracellular amplification distinguish saRNA. Longer expression does not establish greater physicochemical stability; integrity and encapsulation need formulation-specific evaluation [58].
Trans-amplifying RNA (taRNA)	A common design combines non-replicating replicase mRNA with an antigen-containing trans-replicon lacking a complete functional replicase ORF. Same-cell delivery enables amplification in trans through retained cis-acting elements [59].	Replicase and antigen modules can be optimized independently, and antigen cassettes can be exchanged readily. Shortening the antigen RNA may partially mitigate full-length IVT yield problems associated with a single long saRNA transcript, and the replicase-to-antigen RNA ratio can be adjusted.	Same-cell delivery and the replicase-to-trans-replicon RNA ratio are critical. Each RNA would require separate assessment of identity, purity, and integrity, while lipid-based formulations would require RNA-specific assessment of encapsulation, ratio, and functional co-delivery. Co-formulation alone does not demonstrate co-loading in every particle; product-specific functional trans-amplification should therefore be characterized. Clinical and regulatory precedents remain limited.	taRNA may improve antigen modularity and address the long single-transcript problem of saRNA, but delivery-system design and CMC can become even more complex.
Circular RNA (circRNA)	A covalently closed RNA lacking free 5′ and 3′ ends. It generally does not depend on a 5′ cap or poly(A) tail and uses an internal ribosome entry site, an m6A-related element, or another engineered translation element for cap-independent translation. It does not undergo replicase-mediated RNA amplification.	Resistance to exonuclease-mediated degradation may improve RNA stability and support sustained translation. The absence of a viral replicase avoids theoretical concerns related to replicase expression, alphavirus backbones, and recombination, and reduces dependence on a capping process.	Key CMC challenges include circularization efficiency and removal of residual linear precursors, nicked or concatemeric RNA, and intron/ligation by-products. Expression varies with the translation element; application to large ORFs and standardization of topology-specific purity assays remain limited. Human vaccine and licensing evidence is sparse relative to linear mRNA and saRNA.	circRNA is differentiated by template stability and prolonged translation rather than increased RNA copy number. Its stability potential is substantial, but clinical and regulatory maturity remains low.

Abbreviations: CMC, chemistry, manufacturing, and controls; dsRNA, double-stranded RNA; IRES, internal ribosome entry site; IVT, in vitro transcription; LNP, lipid nanoparticle; nsP, nonstructural protein; ORF, open reading frame; sgP, subgenomic promoter; UTR, untranslated region.

**Table 2:** Clinical-stage and publicly disclosed saRNA/srRNA development landscape (as of 6 May 2026).

Originator/Disclosed Development Partner(s)	Candidate/Platform	Indication/Antigen	Highest Stage/Status (May 2026)	Region/Registry	Ref.
Arcturus Therapeutics; CSL Seqirus; Meiji Seika Pharma; Vinbiocare	ARCT-154 (Kostaive; zapomeran); STARR/LUNAR LNP	SARS-CoV-2	Licensed	Japan, EU, UK; NCT05012943	[15,16,17,18,19,20]
Arcturus Therapeutics; CSL Seqirus; Meiji Seika Pharma	ARCT-2301	SARS-CoV-2 bivalent; ancestral D614G + Omicron BA.4/5	Phase 3	Japan; jRCT2031230340	[22]
Arcturus Therapeutics; CSL Seqirus	ARCT-2303	SARS-CoV-2 Omicron	Phase 3	Multinational; NCT06279871	[23]
Gennova Biopharmaceuticals; HDT Bio; Emcure	GEMCOVAC-19; LION/repRNA	SARS-CoV-2	Authorized/EUA	India; regulatory filings	[27]
Gennova Biopharmaceuticals	GEMCOVAC-OM	SARS-CoV-2 Omicron	Authorized/EUA	India; CTRI/2022/10/046475	[24,28]
Imperial College London; VaxEquity	LNP-nCoVsaRNA (COVAC1)	SARS-CoV-2	Phase 2a	UK, EU; ISRCTN17072692; EudraCT 2020-001646-20	[25,26]
MRC/UVRI and LSHTM Uganda	LNP-nCOV saRNA-02	SARS-CoV-2	Phase 1	Uganda; NCT04934111	[45,46]
VLP Therapeutics Japan	VLPCOV-02; 5mC-modified saRNA	SARS-CoV-2	Phase 1/2	Japan; jRCT2051230005	[30,73]
VLP Therapeutics Japan	VLPCOV-04	SARS-CoV-2; Omicron XBB.1.5 booster	Phase 3	Japan; jRCT2051230141	[29]
HDT Bio; Azidus; MCTI-CIMATEC	HDT-301; LION/repRNA	SARS-CoV-2	Phase 1	United States, Brazil; NCT05132907; NCT04844268	[48,49,50]
AAHI; ImmunityBio	AAHI-SC2; AAHI-SC3; saRNA-NLC	SARS-CoV-2 booster	Phase 1/2	South Africa; NCT05370040	[40]
Gritstone Bio	GRT-R910	SARS-CoV-2; spike + conserved non-spike T-cell epitopes	Phase 1	UK; NCT05148962	[41]
Gritstone Bio; CEPI	GRT-R912/GRT-R914/GRT-R918	SARS-CoV-2; variant/non-spike T-cell epitope candidates	Phase 1	South Africa; NCT05435027	[42]
Arcturus Therapeutics; CSL Seqirus	ARCT-2138	Seasonal influenza multivalent	Phase 1	Australia; NCT06125691	[33,34]
Arcturus Therapeutics; CSL Seqirus; BARDA	ARCT-2304 (LUNAR-H5N1)	Pandemic influenza A(H5N1)	Phase 1	United States; NCT06602531	[35,36]
Pfizer; BioNTech	PF-07852352/saRNA influenza vaccine preparations	Seasonal influenza	Phase 1	United States; NCT05227001	[31,32]
CSL Seqirus	Investigational saRNA influenza vaccine; candidate name variably disclosed	Influenza; seasonal/pandemic program	Phase 1	Australia; NCT06028347	[37,38]
GSK	RG-SAM	Rabies virus	Phase 1	United States; NCT04062669	[47]
Replicate Bioscience	RBI-4000; srRNA	Rabies virus	Phase 1	United States; NCT06048770	[11,43,44]
HDT Bio	HDT-321	Crimean–Congo hemorrhagic fever virus	Phase 1	United States; NCT06799013	[39]
Gritstone Bio	GRANITE regimen; ChAd68 prime + saRNA boost	Personalized neoantigen cancer vaccine	Phase 1/2	United States; NCT03639714	[52]
Gennova Biopharmaceuticals; CEPI	Nipah saRNA vaccine candidate	Nipah virus	Preclinical/public program	No clinical registry identified	[51]

Abbreviations: 5mC, 5-methylcytosine; AAHI, Access to Advanced Health Institute; BARDA, Biomedical Advanced Research and Development Authority; CEPI, Coalition for Epidemic Preparedness Innovations; ChAd68, chimpanzee adenovirus serotype 68; CIMATEC, Centro Integrado de Manufatura e Tecnologia; CTRI, Clinical Trials Registry–India; EU, European Union; EUA, emergency use authorization; EudraCT, European Union Drug Regulating Authorities Clinical Trials Database; LION, lipid inorganic nanoparticle; LNP, lipid nanoparticle; LSHTM, London School of Hygiene & Tropical Medicine; MCTI, Brazilian Ministry of Science, Technology and Innovation; MRC, Medical Research Council; NLC, nanostructured lipid carrier; repRNA, replicon RNA; saRNA, self-amplifying RNA; srRNA, self-replicating RNA; UK, United Kingdom; UVRI, Uganda Virus Research Institute. Notes: Status as of [May 2026]; subject to change. Clinical status was compiled from ClinicalTrials.gov, jRCT/CTRI records, regulatory assessments, company announcements, and peer-reviewed literature. srRNA, repRNA, and saRNA are treated as members of the saRNA family when they use a self-amplifying replicon mechanism. “Licensed” denotes full marketing authorization; “Authorized/EUA” denotes emergency use authorization. Entities are listed according to their publicly disclosed roles in the cited clinical or regulatory program as of the cutoff date; inclusion does not necessarily indicate current ownership, licensing, or commercial rights.

**Table 3:** Regulatory issues specific to saRNA identified in assessments of Kostaive across regulatory agencies.

Regulatory Issue	Japan: PMDA [17]	European Union: EMA [16]	United Kingdom: MHRA [20,69]
Review pathway and scope	Reviewed under the standard approval pathway for a drug containing a new active ingredient, with priority review and pre-assessment consultation. Following an independent review, product-specific approval conditions and an 8-year re-examination period were established.	The standard centralized review pathway was applied. Zapomeran (ARCT-154) ^a^ was recognized as a new active substance and placed under additional monitoring.	International Recognition Procedure Route B was applied, with the EMA as the reference regulator. EMA conclusions were considered, while saRNA-specific nonclinical and clinical issues were examined through targeted assessment.
Platform/formulation and quality comparability	ARCT-021 ^b^ data were used because ARCT-021 and ARCT-154 share the same LNP composition and manufacturing process and differ primarily in antigen sequence. Comparability of drug substance and drug product quality attributes before and after manufacturing changes was also assessed.	ARCT-021 and ARCT-154 were considered chemically and physically similar platform products; ARCT-021 challenge, tissue-distribution, and reproductive-toxicity data were accepted and supplemented with direct ARCT-154 data. Comparability between the liquid-frozen clinical formulation and the lyophilized commercial formulation was demonstrated through manufacturing-process comparisons, batch-release testing, and extended characterization.	ARCT-021 surrogate data and bridging from liquid ARCT-021 to lyophilized ARCT-154 were explicitly accepted. On the basis of the EMA quality assessment, the public report confirmed the adequacy of GMP and quality data.
Biodistribution and persistence of self-amplifying RNA	LUC-saRNA encoding replicase and luciferase produced higher injection-site concentrations than conventional mRNA through day 8; both RNAs were nearly undetectable by days 15–30. Extremely prolonged replicon persistence or prolongation of adverse reactions was considered unlikely; information for healthcare professionals and vaccine recipients was required to reflect the mechanism and potential persistence.	Local exposure to saRNA was recognized as potentially greater and longer than with conventional mRNA; however, ARCT-021 biodistribution data in mice indicated that mRNA was present in plasma and tissues for only approximately two weeks, and the EMA considered long-term exposure to the active substance not to be a concern	At the MHRA’s request, the direct ARCT-154 biodistribution study ARC24-052 ^e^ was additionally reviewed. Negative-strand RNA and nsP1 were low and transient; genomic RNA largely cleared, with only trace detection in one of ten animals at the injection site on day 60. Overall findings supported self-limiting amplification.
Novel LNP lipid ATX-126	ATX-126 ^d^ was evaluated as a novel excipient and potassium sorbate as an excipient newly used by the intramuscular route. Safety concerns at the clinical regimen were considered unlikely, but acceptance of these excipients was not to be regarded as a precedent for future products.	The review focused on prolonged tissue persistence, ovarian exposure, reproductive toxicity, and genotoxicity read-across. The risk was accepted based on a 27-fold rabbit-to-human safety margin, the absence of correlated reproductive-organ toxicity, and data from related lipids and platforms.	Additional physicochemical and compositional data were requested to determine whether the ARCT-810 ^c^/ATX-95 ^d^ genotoxicity package could be applied to ATX-126. Genotoxic risk was considered low in view of high animal exposure margins, intermittent clinical dosing, and the absence of correlated toxicological findings.
Replicase- and recombination-related risks	The VEEV structural genes were confirmed to be replaced by the spike sequence, preventing infectious-particle production. The absence of reverse transcriptase was considered to make integration into host–cell DNA unlikely. No separate detailed assessment of recombination or routine recombinant-virus surveillance was identified in the public PMDA report.	Theoretical recombination with a wild-type VEEV-like virus was considered unlikely based on superinfection exclusion, cytoplasmic localization, codon optimization, and replicase mutations expected to attenuate pathogenicity if recombination occurred. Potential effects of pre-existing or induced anti-replicase T-cell immunity and concomitant antiviral or interferon treatment on replicase activity remained follow-up issues.	Additional questions were raised regarding recombination, replicase specificity, and replicase immunogenicity. Although the theoretical risk could not be excluded, it was considered low; specificity for Kostaive cis-acting elements was also considered to make amplification of a co-administered mRNA vaccine unlikely.
Risk management plan and postauthorization measures	Important identified risk: shock/anaphylaxis. Important potential risks: myocarditis/pericarditis, VAED/VAERD, and Guillain–Barré syndrome. Important missing information: pregnancy and lactation. Required measures included early postmarketing vigilance, a general use-results survey in 1250 individuals, follow-up data from ARCT-154-J01, prompt submission of ongoing Japanese clinical results, and written informed consent based on current information.	No important identified risk was specified. Important potential risks were myocarditis/pericarditis and thromboembolic events. Missing information included pregnancy/lactation, immunocompromised populations, autoimmune/inflammatory diseases, coadministration with other vaccines, and long-term safety; postauthorization safety assessment and studies in special populations were planned.	The RMP and pharmacovigilance system were accepted, and thromboembolism was retained as an important potential risk. No myocarditis/pericarditis occurred in clinical development, but a class warning was included. Submission of neutralization data against circulating variants was required by June 30, 2026; review of Japanese postmarketing data identified no new safety signal at the time assessed.

^a^ Kostaive is a lyophilized sa-mRNA–LNP COVID-19 vaccine containing 5 μg/dose of ARCT-154 (active substance: zapomeran). ^b^ ARCT-021 is a first-generation predecessor saRNA candidate encoding ancestral Wuhan SARS-CoV-2 spike. It uses the same LNP and core RNA elements as ARCT-154 and supported nonclinical and clinical bridging. ^c^ ARCT-810 is an mRNA therapeutic in clinical development that encodes human ornithine transcarbamylase and shares certain LNP lipid components with ARCT-154. ^d^ ATX-126 is a novel ionizable lipid used in the Kostaive LNP; ATX-95 is a structurally related cationic lipid used in ARCT-810 and was used for genotoxicity read-across to ATX-126. ^e^ ARC24-052 is a single-dose mouse nonclinical study directly evaluating ARCT-154 biodistribution and persistence. ARCT-154-J01 is a phase 3 noninferiority immunobridging study comparing the immunogenicity and safety of a Kostaive booster with Comirnaty in Japanese adults. Abbreviations: DNA, deoxyribonucleic acid; EMA, European Medicines Agency; GMP, good manufacturing practice; IRP, International Recognition Procedure; LNP, lipid nanoparticle; LUC-saRNA, luciferase-encoding self-amplifying RNA; MHRA, Medicines and Healthcare products Regulatory Agency; mRNA, messenger RNA; nsP1, nonstructural protein 1; PMDA, Pharmaceuticals and Medical Devices Agency; RMP, risk management plan; RNA, ribonucleic acid; saRNA, self-amplifying RNA; VAED, vaccine-associated enhanced disease; VAERD, vaccine-associated enhanced respiratory disease; VEEV, Venezuelan equine encephalitis virus. Sources: EMA Assessment Report [16], PMDA Report on the Deliberation Results [17], and MHRA Public Assessment Report and official approval announcement [20,69].

## Data Availability

No new data were created or analyzed in this study. Data sharing is not applicable to this article.

## Abbreviations

The following abbreviations are used in this manuscript:

AAHI	Access to Advanced Health Institute
APC	antigen-presenting cell
BARDA	Biomedical Advanced Research and Development Authority
CCHFV	Crimean–Congo hemorrhagic fever virus
CEPI	Coalition for Epidemic Preparedness Innovations
circRNA	circular RNA
CMC	chemistry, manufacturing, and controls
CQA	critical quality attribute
CRP	C-reactive protein
dsRNA	double-stranded RNA
DSPC	1,2-distearoyl-sn-glycero-3-phosphocholine
EMA	European Medicines Agency
EUA	Emergency use authorization
FDA	Food and Drug Administration
GMP	good manufacturing practice
ICH	International Council for Harmonisation
IFN	interferon
IRES	internal ribosome entry site
IRP	International Recognition Procedure
IVT	in vitro transcription
LNP	lipid nanoparticle
M1	matrix protein 1
MAP	microneedle array patch
MDA5	melanoma differentiation-associated protein 5
MHC	major histocompatibility complex
MHRA	Medicines and Healthcare products Regulatory Agency
NHP	nonhuman primate
NLC	nanostructured lipid carrier
NOAEL	no-observed-adverse-effect level
NP	nucleoprotein
nsP	nonstructural protein
OAS	2′-5′-oligoadenylate synthetase
ORF	open reading frame
PDI	polydispersity index
PEG	polyethylene glycol
PKR	protein kinase R
PMDA	Pharmaceuticals and Medical Devices Agency
RBD	receptor-binding domain
RdRp	RNA-dependent RNA polymerase
RIG-I	retinoic acid-inducible gene I
RMP	risk management plan
RT-ddPCR	reverse-transcription droplet digital polymerase chain reaction
saRNA	self-amplifying RNA
SFV	Semliki Forest virus
sgP	subgenomic promoter
srRNA	self-replicating RNA
taRNA	trans-amplifying RNA
Th1	T helper 1
TLR	Toll-like receptor
UTR	untranslated region
VAED	vaccine-associated enhanced disease
VAERD	vaccine-associated enhanced respiratory disease
VEEV	Venezuelan equine encephalitis virus
WHO	World Health Organization
